# Intermittent MEK inhibition for the treatment of metastatic uveal melanoma

**DOI:** 10.3389/fonc.2022.975643

**Published:** 2022-09-29

**Authors:** Shaheer Khan, Sapna P. Patel, Alexander N. Shoushtari, Grazia Ambrosini, Serge Cremers, Shing Lee, Lauren Franks, Shahnaz Singh-Kandah, Susana Hernandez, Naomi Sender, Kristina Vuolo, Alexandra Nesson, Prabhjot Mundi, Benjamin Izar, Gary K. Schwartz, Richard D. Carvajal

**Affiliations:** ^1^ Department of Medicine Columbia University Irving Medical Center, New York, NY, United States; ^2^ Department of Melanoma Medical Oncology University of Texas MD Anderson Cancer Center, Houston, TX, United States; ^3^ Department of Medicine Memorial Sloan Kettering Cancer Center, New York, NY, United States; ^4^ Mailman School of Public Health, Columbia University, New York, NY, United States

**Keywords:** uveal melanoma, metastatic, MEK inhibition, intermittent dosing, selumetinib

## Abstract

**Introduction:**

Uveal melanoma (UM) is associated with poor outcomes in the metastatic setting and harbors activating mutations resulting in upregulation of MAPK signaling in almost all cases. The efficacy of selumetinib, an oral allosteric inhibitor of MEK1/2, was limited when administered at a continual dosing schedule of 75 mg BID. Preclinical studies demonstrate that intermittent MEK inhibition reduces compensatory pathway activation and promotes T cell activation. We hypothesized that intermittent dosing of selumetinib would reduce toxicity, allow for the administration of increased doses, and achieve more complete pathway inhibition, thus resulting in improved antitumor activity.

**Methods:**

We conducted a phase Ib trial of selumetinib using an intermittent dosing schedule in patients with metastatic UM. The primary objective was to estimate the maximum tolerated dose (MTD) and assess safety and tolerability. Secondary objectives included assessment of the overall response rate (RR), progression-free survival (PFS) and overall survival (OS). Tumor biopsies were collected at baseline, on day 3 (on treatment), and between days 11-14 (off treatment) from 9 patients for pharmacodynamic (PD) assessments.

**Results:**

29 patients were enrolled and received at least one dose of selumetinib across 4 dose levels (DL; DL1: 100 mg BID; DL2: 125 mg BID; DL3: 150 mg BID; DL4: 175 mg BID). All patients experienced a treatment-related adverse event (TRAE), with 5/29 (17%) developing a grade 3 or higher TRAE. Five dose limiting toxicities (DLT) were observed: 2/20 in DL2, 2/5 in DL3, 1/1 in DL4. The estimated MTD was 150 mg BID (DL3), with an estimated probability of toxicity of 29% (90% probability interval 16%-44%). No responses were observed; 11/29 patients achieved a best response of stable disease (SD). The median PFS and OS were 1.8 months (95% CI 1.7, 4.5) and 7.1 months (95% CI 5.3, 11.5). PD analysis demonstrated at least partial pathway inhibition in all samples at day 3, with reactivation between days 11-14 in 7 of those cases.

**Conclusions:**

We identified 150 mg BID as the MTD of intermittent selumetinib, representing a 100% increase over the continuous dose MTD (75 mg BID). However, no significant clinical efficacy was observed using this dosing schedule.

## Introduction

Uveal melanoma (UM) is a rare malignancy arising from melanocytes within the uveal tract with a high propensity for hematogenous metastatic spread despite effective treatment of the primary tumor (1). Despite the recent approval by the United States Food and Drug Administration of tebentafusp for HLA*A0201 positive patients with metastatic uveal melanoma, clinical outcomes remain poor and there remain no FDA approved therapies for HLA*A0201 negative patients or for those with UM refractory to treatment with tebentafusp (2).

UM is characterized by constitutive activation of the G-protein-α signaling pathway *via* mutations in GNAQ, GNA11, PLCB4 or CYSLTR2 which are present in a mutually exclusive pattern in virtually all cases (3, 4). These mutations result in activation of downstream pathways including the mitogen-activated protein kinase (MAPK) pathway, amongst others (5). Several clinical trials of various inhibitors of the MAPK pathway, including the MEK inhibitors selumetinib and trametinib as well as the ERK inhibitor ulixertinib, have been performed, with limited efficacy observed (6). A randomized phase II trial of selumetinib 75 mg BID compared to chemotherapy with temozolomide or DTIC demonstrated an improvement in median PFS from 7 to 16 weeks in favor of selumetinib, with 14% of patients achieving an objective radiographic response (7). Although SUMIT, the subsequent randomized placebo controlled phase III trial of selumetinib 75 mg BID combined with dacarbazine demonstrated no improvement in PFS when compared with dacarbazine, SELPAC, a randomized phase II study phase of selumetinib alone or in combination with paclitaxel met its primary endpoint of improved PFS for the combination of selumetinib 75 mg BID combined with paclitaxel when compared with selumetinib alone, with a median PFS of 4.8 months and 3.4 months, respectively (8–10).

Continuous exposure to RAF or MEK inhibitors in preclinical models of BRAF mutant melanoma results in the development of feedback reactivation of the ERK pathway within tumor cells and subsequent resistance to treatment (11, 12). Immunologically, inhibition of MEK signaling reduces the proliferation of T cells *in vitro* and prolonged blockade of T cell receptor signaling by MEK inhibitors interferes with effector function and proliferation at the tumor site (13–15). Cyclical pulsatile dosing of MEK inhibition, on the other hand, maintains T cell activation and proliferation and results in the abrogation of feedback activation of the ERK pathway within tumors cells observed with continual exposure (11, 12, 16).

We therefore hypothesized that intermittent MEK inhibition would result in clinical efficacy greater than that achieved with continual inhibition in metastatic uveal melanoma. To test this hypothesis, we developed and conducted this multicenter, single arm, open-label, phase Ib study of selumetinib administered using a pulsatile dosing schedule.

## Materials and methods

### Study design

This was a multi-center, open-label, phase Ib study conducted at Columbia University Irving Medical Center (New York, NY), University of Texas MD Anderson Cancer Center (Houston, TX), and Memorial Sloan Kettering Cancer Center (New York, NY). The primary objective of this study was to determine the maximum tolerated dose (MTD) of selumetinib administered using an intermittent dosing schedule in patients with metastatic UM. Secondary objectives were to evaluate the efficacy of intermittent selumetinib as determined by investigator-assessed overall response rate (ORR) using RECIST v1.1 criteria, progression free survival (PFS), and overall survival (OS). Exploratory objectives included pharmacodynamic (PD) assessment of target inhibition of the MEK pathway.

The protocol was approved by the institutional review board at each participating institution and conducted under the principles of the International Council of Harmonization and Good Clinical Practice. Drug and funding for this investigator sponsored research study was provided by AstraZeneca Inc (Wilmington, DE, USA) to support the study. AstraZeneca had no role in data collection, analysis, or interpretation, or in writing this report. This study was registered with www.clinicaltrials.gov as NCT02768766.

Eligible patients received selumetinib orally twice daily on days 1-3, 8-10, 15-17, and 22-24 of each 28-day cycle at approximately the same time each day. Given the mean half-life of selumetinib of 5-8 hours, the four-day off schedule was thought to be sufficient for complete drug washout. Furthermore, this schedule was postulated to limit the reactivation of MAPK signaling which had been observed pre-clinically to reach steady state levels after 48-72 hours of continuous treatment. Six dose levels (DL) of selumetinib were investigated however only 4 dose levels were administered: DL1 – 100 mg BID; DL2 – 125 mg BID; DL3 – 150 mg BID; and, DL4 – 175 m BID. The MTD was estimated using the time to event continual reassessment method (TITE-CRM) with a target DLT rate of 0.25 (17). TITE-CRM was used as it allows use of partial information before a complete follow-up is achieved and allows for a longer toxicity evaluation window beyond the first cycle to account for late onset toxicities. As a result, trials can be conducted in a continuous fashion. An empirical dose-toxicity model was used with a prior mean of 0 and variance of 1.34, calibrated to an indifference interval such that the method would select a dose that yields a rate of DLT between 18% and 32% based on the MTD being defined as the dose associated with a target probability of dose limiting toxicity of 0.25. Based on prior clinical data, the estimated MTD prior to conduct of the trial was DL2. The sample size of 28 was obtained based on the operating characteristics simulating various toxicity scenarios which yielded at least a 58% probability of selecting the correct dose. The design did not allow for dose skipping or escalation immediately after a DLT. All patients who received at least one dose of selumetinib were evaluable for toxicity. However, patients who did not complete 75% of dosing due to reasons other than treatment related toxicity (e.g. non-compliance) or who discontinued due to reasons other than treatment related toxicity (i.e. progression or death) prior to completing 4 cycles of therapy without having experienced a DLT were replaced. Partial toxicity information while greater than 75% compliant was included for dose assignments and the estimation of the MTD.

Descriptive statistics were used to report baseline characteristics of the study population. The observed rate and estimated rate of DLT were reported for each dose level along with the 90% probability interval. The number and percentage of patients with adverse events were summarized taking the highest grade and reported by grade for AEs that were at least possibly related to treatment. AEs with at prevalence of least 10% were reported separately. The overall response rate (ORR) was defined as a confirmed complete response (CR) or partial response (PR) according to RECIST 1.1 criteria (18) and reported by dose level. Disease control rate (DCR) was defined as the percentage of patients with a confirmed CR, PR or stable disease. PFS was measured from date of enrollment until the date of progression or death, whichever occurred first. OS was defined as the time from the date of registration to the date of death by any cause.

### Patient selection

Eligible patients were aged 18 years or older and had histologically confirmed unresectable metastatic UM. Patients may have been treated with any number of prior therapies for metastatic disease; however, prior MEK inhibitor therapy was not permitted. An Eastern Cooperative Oncology Group (ECOG) (8) performance status of ≤2 and adequate organ function were required. Exclusion criteria included patients with active brain or spinal cord metastases, known cardiac conditions placing them at higher risk of cardiac toxicity, or history of retinal detachment or retinal vein occlusion. All participating patients provided written informed consent before enrollment.

### DLT definition

Toxicity was graded using National Cancer Institute Common Terminology Criteria for Adverse Events (CTCAE v. 4.0). For recurrent adverse events (AEs), the highest reported grade per event per patient was assessed. The DLT observation period was 8 weeks from the initiation of treatment. DLT was defined as any of the following: 1) any serious AE deemed related to the investigational treatment (included grade 1 or 2 ocular toxicity requiring dose reduction); 2) receiving <75% of the planned doses during weeks 1-8; and, 3) death related to the investigational regimen.

### Evaluation of clinical activity

Imaging was obtained at baseline, at 8 weeks after start of study treatment, and then every 8 weeks thereafter. Imaging of the chest, abdomen, and pelvis was required at each time point, with contrast enhanced MRI of the liver performed whenever possible. Response was assessed using RECIST v.1.1 criteria by investigator assessment. Criteria for removal from study included radiographic or clinical disease progression, or unacceptable toxicity.

### Pharmacokinetic (PK) analysis

Blood samples were collected pre-dose and at 0, 0.5, 1, 2, 3, 4 and 8 hours after selumetinib dosing on day 1 and day 2, and pre-dose and at 0, 0.5, 1, 2, 3 and 4 hours on days 15 and 17 of the first cycle. Selumetinib and N-desmethyl-selumetinib were measured in serum using liquid chromatography – tandem mass spectrometry (Labcorp Bioanalytical Services, Princeton, NJ). The lower limit of quantification of the assay was 2.0 ng/mL for selumetinib and its metabolite. Intra- and inter-day accuracy and precision of the assay were <15% for both analytes. Non-compartmental analyses were performed on the data for day 1, 2, 15 and 17 using Winnonlin software (Phoenix Winnonlin 8.3.1.5014, Certara L.P., Princeton, NJ). Parameters calculated were Tmax, Cmax, t½, AUC0-4h, AUC0-8h and CL. Data were summarized per dose level and per occasion. Dose-exposure relationships were explored by plotting dose versus the various parameters. Accumulation was investigated using the AUC assessed at various days.

### Pharmacodynamic (PD) analysis

Serial tumor biopsies were collected from patients at baseline, on day 3, and between days 11 and 14. Flash-frozen specimens were lysed in RIPA lysis buffer. Equal amounts of protein were loaded on 4-20% PAGE gels (Invitrogen) and analyzed by immunoblotting using antibodies for pERK, ERK, cyclin D1 and α-tubulin (Cell Signaling). Signals from secondary antibodies were detected using ECL (Pierce) and autoradiography films. Densitometry analysis of each band was performed using Photoshop C6 and normalized to tubulin expression.

### Pharmacokinetic-pharmacodynamic (PK-PD) and systemic exposure-outcome analysis

PK-PD relationships were investigated by plotting Cmax, AUC0-4h and AUC0-8h versus percent decrease in ERK phosphorylation. In addition, recovery from ERK phosphorylation was plotted against Cmax, AUC0-4h, AUC0-8h as well as C0h collected on day 15 of cycle 1. Systemic exposure – outcome relationships were investigated by plotting Cmax and the average AUC0-4h versus PFS, OS and disease progression.

## Results

### Patient demographics

Between April 2017 and June 2020, 29 patients were enrolled and received at least one dose of selumetinib. The median age was 58 years old (range, 30-85), 52% were female, and 83% were white. Most patients were previously treated, with 66% having received prior immunological checkpoint blockade and 31% having received 2 or more prior systemic therapies. Forty-eight percent of patients had an LDH above the upper limit of normal. Baseline characteristics are shown in **Table 1**.

**Table 1:** Baseline characteristics.

Baseline Characteristics	
**Age, median** (range)	58 years (30-85)
18-49 years (%)	6 (21%)
50-69 years (%)	20 (69%)
≥70 years (%)	3 (10%)
**Sex**	
Male (%)	14 (48%)
Female (%)	15 (52%)
**Race**	
White (%)	21 (72.4%)
Other/Unknown (%)	8 (27.6%)
**Prior systemic treatments, median** (range)	1 (0-5)
0	9 (31%)
1	7 (24%)
≥2	9 (31%)
Prior immune checkpoint blockade	19 (66%)
**LDH. median** (range)	321 (163-1028)
Normal (%)	13 (45%)
Elevated (%)	14 (48%)
>2x Normal (%)	3 (10%)
Unknown (%)	2 (7%)
**GNAQ/GNA11 mutation status**	
GNAQ (%)	13 (45%)
GNA11 (%)	6 (21%)
Unknown (%)	10 (34%)
**Sites of metastases**	
Hepatic only (%)	4 (14%)
Extra-hepatic only (%)	3 (10%)
Hepatic and extra-hepatic (%)	22 (76%)
**Uveal Melanoma AJCC M Staging**	
M1a (%)	17 (59%)
M1b (%)	9 (31%)
M1c (%)	3 (10%)

### Adverse events and MTD determination

Three patients were treated at DL1 (100 mg BID); 20 at DL2 (125 mg BID); 5 at DL3 (150 mg BID); and 1 at DL4 (175 mg BID). All patients experienced at least one AE related to therapy of any grade, with 5 patients (17%) experiencing a grade 3 or higher AE, including one patient who developed a grade 4 anemia (**Table 2**). The most frequently observed treatment related AEs were fatigue, rash, nausea/diarrhea, and anemia. The most common grade 3 AE was transaminase elevation. Six patients (21%) required dose reduction for treatment-related toxicity. Five patients discontinued therapy due to toxicity. Toxicities associated with treatment discontinuation were liver enzyme elevation (n=2), retinopathy (n=2), and nausea/weight loss.

**Table 2:** Adverse events (all grades, occurring in ≥10% of patients) possibly, probably or definitely related to study treatment.

	Grade 1 n(%)	Grade 2 n(%)	Grade 3 n(%)	Grade 4 n(%)	All Grades*
**Laboratory**					
Anemia	11 (38)	1 (3)	0	1 (3)	13 (45%)
Increased AST/ALT	10 (34)	0	2 (7)	0	12 (41%)
Decreased WBC	5 (17)	2 (7)	1 (3)	0	8 (28%)
**Gastrointestinal**					
Nausea	12 (41)	5 (17)	0	0	17 (59%)
Diarrhea	14 (48)	2 (7)	0	0	16 (55%)
Abdominal pain	5 (17)	4 (14)	3 (10)	0	12 (41%)
Constipation	8 (28)	2 (7)	0	0	10 (34%)
Vomiting	5 (17)	3 (10)	0	0	8 (28%)
**Other**					
Fatigue	19 (66)	6 (21)	0	0	25 (86%)
Rash acneiform	14 (48)	5 (17)	0	0	19 (65%)
Hypertension	3 (10)	9 (31)	2 (7)	0	14 (48%)
Pruritus	10 (34)	0	0	0	10 (345)
Retinopathy/Blurred Vision	8 (28)	0	0	0	8 (28%)

*Total column indicates how many patients experienced that AE. If a patient experienced an AE, they are categorized in the highest grade.

No DLTs were observed on DL1 (**Table 3**). Two patients on DL2 (grade 3 hypertension and grade 3 abdominal pain) and two patients on DL3 experienced DLTs (grade 3 abdominal pain and grade 3 AST elevation). The only patient treated on DL4 also developed a DLT (grade 3 AST elevation). The second DLT event on DL3 was only identified after one patient was treated on DL4. Due to 2/4 patients in DL3 and 1/1 patients in DL4, the initial MTD was identified as DL2 and subsequent patients received that dose. After accrual of a total of 20 patients to DL2 with identification of only 2 DLT events, one additional patient was enrolled on DL3. This patient did not experience a DLT, thus DL3 was declared the MTD, with an estimated probability of toxicity of 29% (90% probability interval 16%-44%).

**Table 3:** Dose Limiting Toxicities by Dose Level.

	Total patients in dose level	ObservedDLTs	DLT Information
**Dose Level 1 (100 mg BID)**	3	0	
**Dose Level 2 (125 mg BID)**	20	2	Grade 3 Hypertension Grade 3 Abdominal pain
**Dose Level 3 (150 mg BID)**	5	2	Grade 3 Abdominal pain Grade 3 AST elevation
**Dose Level 4 (175 mg BID)**	1	1	Grade 3 AST elevation
**All**	29	5	

### Clinical efficacy

**Figure 1:**
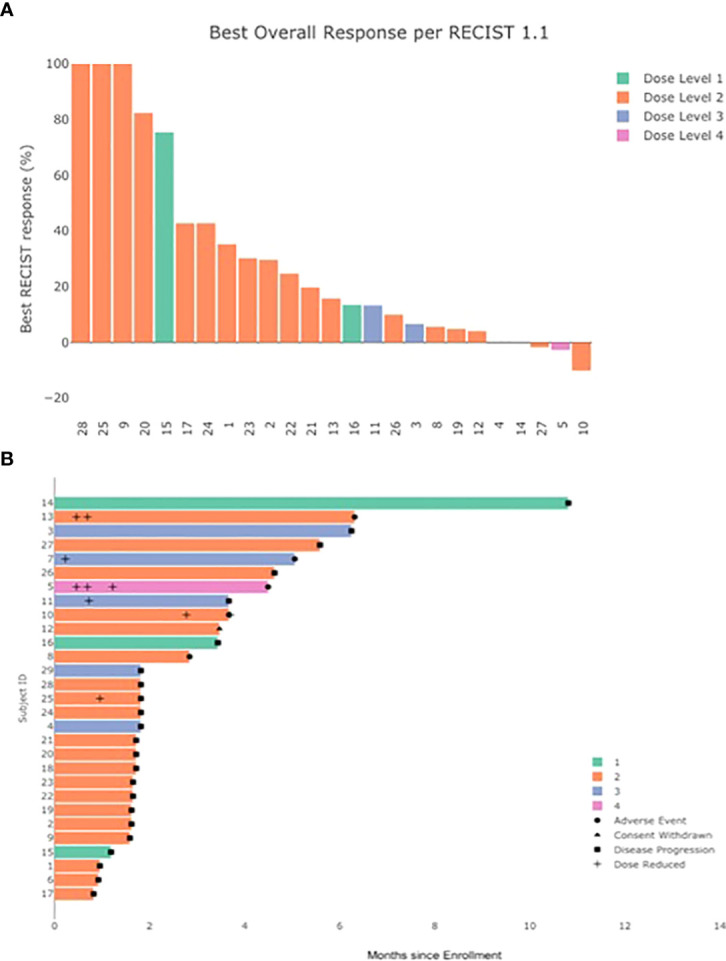
Clinical response to treatment. **(A)** Best % change in sum of target lesions by investigator assessed RECIST v1.1 (by dose level) **(B)** duration of treatment and time to adverse event, dose reduction, and disease progression.

All 29 patients were evaluable for response (**Table 4**). No patients demonstrated a complete or partial response to therapy by RECIST v1.1 criteria (**Figure 1**). A best response of stable disease was observed in 38% of patients (n=11) at 8 weeks from treatment initiation. The median PFS was 1.8 months (95% CI, 1.7, 4.5) and median OS 7.1 months (95% CI 5.3, 11.5) in the total population. No patients died while receiving study therapy, though 4 patients died within 30 days of treatment discontinuation, all from progression of disease.

**Table 4:** Investigator assessed RECIST v1.1 response by Dose Level.

	Level 1	Level 2	Level 3	Level 4	All Levels
**Complete Response (CR)**	0	0	0	0	0
**Partial Response (PR)**	0	0	0	0	0
**Stable Disease (SD)**	2/3 (66.7%)	6/20 (30.0%)	2/5 (40.0%)	1/1 (100%)	11/29 (38.0%)
**Progressive Disease (PD)**	1/3 (33.3%)	14/20 (70.0%)	3/5 (60.0%)	0	17/29 (62.0%)
**Overall Response Rate (ORR)**	0	0	0	0	0
**Median Time to Progression (range)**	3.4 months (1.2-10.8)	1.7 months (0.9-5.5)	3.7 months (0.9-6.2)	Not Applicable	–
**Median duration of Stable Disease**	7.1 months	4.1 months	4.9 months	Not Applicable	–

### Pharmacokinetic analysis: selumetinib concentration over time

**Figure 2:**
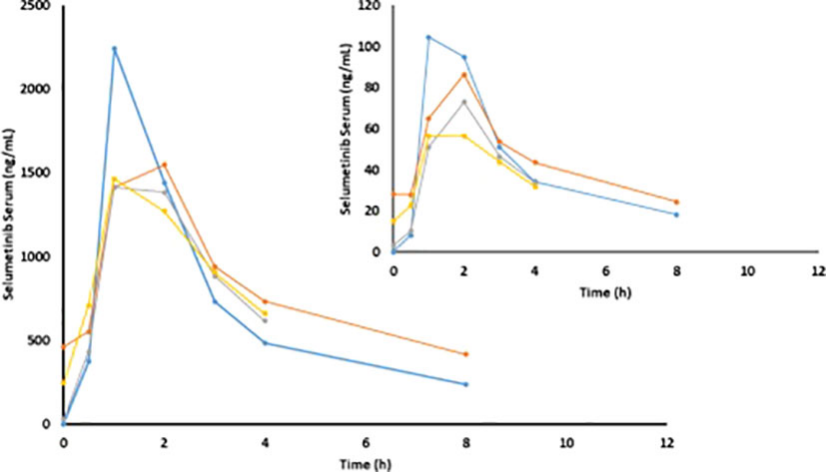
Mean selumetinib and N-desmethyl-selumetinib (insert) concentrations on C1D1 (blue), C1D2 (orange), C1D15 (grey) and C1D17 (yellow) in patients at the 125mg dose level (n=20).

The results of non-compartmental analyses are summarized in **Table 5** and illustrated for the 125mg dose regimen in **Figure 2**. Serum concentrations of selumetinib and its metabolite N-desmethyl-selumetinib increased shortly after oral intake and reached a maximum after approximately 1.7 hours. This was followed by a bi-exponential decline with an average terminal half-life of 4.3h. This half-life, calculated from the 8h serum concentration curves, is likely an underestimation, as selumetinib was quantifiable in serum of nine out of nineteen patients prior to the third three-day course (day 15). Cmax and AUC0-4h and AUC0-8h increased with increasing dose, but PK seemed stable during each three-day course with little to no accumulation during three days of dosing and during repeated three-day courses. Metabolism was stable with a relatively constant ratio between desmethyl-selumetinib and selumetinib serum concentrations and AUCs throughout the study. **Table 5** reveals substantial overall variability in systemic exposure at each dose level. Intra-patient variability was smaller with a coefficient of variation for Cmax and AUC0-8h at the 125mg dose level of 25.3 and 11.3%, respectively.

**Table 5:** Pharmacokinetic analysis of selumetinib (S) and N-desmethyl-selumetinib (NDS).

Dose (mg)	Patients (n)	Agent (S/NDS)	Tmax (h)	Cmax (ng/mL)	AUC0-4h (h*ng/mL)	AUC0-8h (h*ng/mL)	t½ (h)	CL/F (L/h)
**100**	1-2	S	1.7 ± 0.5 (6)	1453 ± 530 (6)	2903 ± 461 (6)	4265 ± 628 (2)	2.4 ± 0.3 (2)	20.9 ± 3.7 (2)
		NDS	2.0 ± 0.6 (6)	77 ± 32 (6)	171 ± 59 (6)	272 ± 34 (2)	2.9 ± 0.4 (2)	–
**125**	15-14	S	1.5 ± 0.8 (56)	2115 ± 798 (56)	4270 ± 1470 (56)	6042 ± 2210 (29)	4.3 ± 5.7 (26)	18.7 ± 8.1 (26)
		NDS	1.7 ± 0.8 (56)	101 ± 44 (56)	218 ± 83 (56)	353 ± 124 (29)	4.3 ± 3.3 (26)	–
**150**	2	SEL	1.5 ± 0.8 (8)	3041 ± 1155 (8)	6073 ± 1540 (8)	6683 ± 729 (4)	7.9 ± 9.1 (3)	14.8 ± 7.6 (8)
		NDS	1.6 ± 0.7 (8)	97 ± 72 (8)	198 ± 119 (8)	264 ± 125 (4)	6.1 ± 5.0 (3)	–

### Pharmacodynamic analysis: MAP kinase pathway inhibition

**Figure 3:**
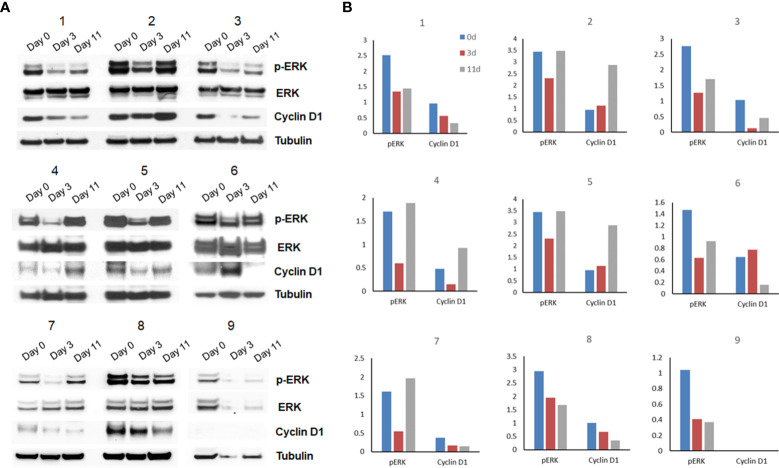
Pharmacodynamic assessment and densitometry analysis (n=9). **(A)** Flash-frozen specimens were lysed and analyzed by immunoblotting using antibodies for pERK, ERK, cyclin D1 and α-tubulin. **(B)** Densitometric quantification of pERK and cyclin D1 expression over time in each patient is shown.

To evaluate target inhibition, phosphorylated ERK, total ERK, and cyclin D1 protein levels were evaluated by Western blot analysis on available serial tumor biopsy specimens collected at baseline, day 3 (on treatment), and between days 11 and 14 (off treatment). Serial samples were collected and available for 9 patients from which the target effects of intermittent selumetinib were assessed. On day 3 of the first cycle, inhibition of MEK pathway signaling was observed in all 9 cases, with a mean reduction in pERK expression of 41.3% (**Figure 3A**). By day 11-14, during the off-treatment window, there was evidence of at least partial pERK recovery in 7 of these cases, resulting in a mean increase in pERK expression of 69.3% from day 3. Densitometric quantification of pERK and cyclin D1 expression over time in each patient is shown in **Figure 3B**. No correlation with degree of ERK, pERK, and cyclin D1 suppression and clinical benefit could be made given the small sample size and overall limited benefit observed in the study.

### Pharmacokinetic-outcome and PK-PD analysis

No correlations were found for AUC0-4h, AUC0-8h and Cmax with PFS or OS. No correlations were found for any of these PK parameters and extent of inhibition of ERK phosphorylation. In addition, no relationship was found between quantitation of selumetinib prior to dose 15 and recovery of ERK phosphorylation on day 11.

## Discussion

This was the first study to assess the intermittent administration of MEK inhibition in patients with metastatic uveal melanoma and was based on preclinical data which identified MEK inhibition as a viable target in uveal melanoma as well as clinical trial data which demonstrated limited benefit with continuous MEK inhibition. This trial was based on the hypothesis that intermittent treatment may be more effective by allowing a higher peak-dose concentration, by inhibiting compensatory activation of alternative pathways, and by maintaining T cell activation and proliferation (16). The results demonstrated that intermittent dosing allowed for a 100% increase in the MTD with a similar toxicity profile compared to previous trials; however, it did not demonstrate any evidence of clinical efficacy as determined by clinical response or survival.

This phase Ib, multicenter, open-label study was performed in 29 patients across 3 cancer centers and exposed patients to 4 different dose levels. A total of five DLT events were recorded which resulted in dose level 3, 150mg twice daily, being identified as the MTD. The most common adverse events included fatigue, rash, nausea, diarrhea, and anemia. The most common grade 3 adverse event was abdominal pain and only one grade 4 event was noted (anemia). The best response across all dose levels was stable disease with a median progression-free response ranging from 1.7-3.7 months by cohort. In the cohort with the highest number of patients (125mg twice daily) the median PFS was 1.7 months and median OS was 7.1 months.

Continuous dosing of selumetinib was previously studied in both a phase II and randomized phase III trial. In the phase II trial, there was improvement in median PFS without OS benefit. This benefit was not seen in the subsequent randomized phase III SUMIT trial which compared selumetinib and dacarbazine to placebo and dacarbazine (8, 9). The results from our study are consistent with the limited benefit observed with continuous selumetinib. Pharmacodynamic, pharmacokinetic, and additional ongoing analyses also suggest that the potential limitations of continuous dosing which were hypothesized to be addressed by intermittent dosing, namely higher peak dose concentration, decreased feedback stimulation, and suppression of immune cell function, were not mechanisms for the lack of benefit seen in the above trials.

Pharmacodynamic assessments performed on serial biopsies obtained from a limited subset of patients (n=9) as part of this study demonstrated a decrease in markers corresponding with MEK inhibition at the initial on-treatment timepoint in all patients; however, 7 of the 9 patients who had reduction in marker expression on-treatment had at least partial recovery during the subsequent off-treatment period. The mean decrease in pERK expression at day 3 in our study approximated that seen on day 14 in the prior randomized phase II study of selumetinib (75mg twice daily). Although it is not clear if suppression of pERK is associated with clinical response, the rapid recovery of pERK in our study may represent a mechanism for the lack of benefit seen in this trial, by allowing tumor regrowth and/or selection of resistant clones in tumors. This hypothesis corresponds with results from a preclinical study in which the MEK inhibitor cobimetinib was intermittently administered in SKMEL28 melanoma cell lines. Treatment interruptions resulted in tumor regrowth that could not be compensated when drug administration was resumed (19). In addition to these preclinical data, the S1320 study, a randomized open-label, phase 2 clinical trial evaluating intermittent dosing of dabrafenib and trametinib in patients with metastatic and unresectable *BRAF* ^V600^ mutant melanoma, found that continuous dosing, rather than intermittent dosing, resulted in a statistically significant improvement in progression-free survival (20). With regard to our hypothesis that intermittent selumetinib may allow for enhanced immune cell function, single nucleus RNA sequencing of serial tumor samples is ongoing and will be reported separately, however preliminary results demonstrated a miniscule population of actively proliferating CD8+ T-cells. While these findings are not clearly associated with selumetinib, it does not appear to suggest any improvement from previous assessments of uveal melanoma liver metastases.

Pharmacokinetic analysis of selumetinib and N-desmethyl-selumetinib concentration over time revealed expected increases in C_max_ and AUC_0-4h_ and AUC_0-8h_ with increasing dose, with little to no accumulation between dosing periods. These values were also increased compared to previously studied continuous dosing schedules of selumetinib (21, 22) and suggest that the higher peak dose concentration allowed by intermittent dosing did not impact tumor response.

There have been no targeted therapies which have demonstrated significant benefit for patients with metastatic UM, despite the identification and exploration of a host of molecular pathways. Molecular targets which have been studied in the metastatic and adjuvant settings without clear evidence of benefit include vascular endothelial growth factor (VEGF), the PI3K/AKT pathway, the MET signaling pathway, as well as heat shock protein 90 (HSP90), MEK 1/2 and protein kinase C (PKC). These negative results, despite preclinical data supporting their importance in propagating growth in UM cells, underscore the need to better understand the biology of this cancer and develop pre-clinical models which more accurately represent the clinical experience. This has also led to several trials assessing combination strategies to improve clinical response, including with MEK inhibition (8, 23, 24). Preliminary data from the SelPac trial, a multicenter randomized study of selumetinib alone or in combination with paclitaxel in 77 patients with metastatic UM demonstrated an improvement in PFS and ORR in the combination arm with a median PFS of 4.8 months (95% CI: 3.8 - 5.6) compared with 3.4 months (95% CI: 2.0 - 3.9) in the selumetinib arm and an ORR of 14% compared to 4% with selumetinib alone. Median OS, however, was similar in both arms at 9 and 10 months, respectively (10). Although the benefit preliminarily noted in this trial is modest and will need to be further validated, it does offer potential for other combination strategies. Targeted agents that are currently being tested in phase 1 and phase 2 trials combined with MEK inhibition include the PKC inhibitor IDE196 (NCT03947385) and the FAK inhibitor IN10018 (NCT04109456).

We acknowledge that our study, which was a small, single-arm, open-label study primarily designed to assess safety and tolerability, is limited in its ability to infer treatment response outcomes. The small number of serial specimens available for correlative analysis also limit potential conclusions that can be drawn from the results available. In addition, given that our trial studied only one combination of intermittent dosing, it is unknown whether an alternative dosing schedule may have had a more pronounced effect. Despite these limitations, our results provide further evidence that MEK inhibition alone, whether administered on a continuous or intermittent basis, is not associated with a significant clinical response and highlight the need for other actionable targets or combination strategies.

In summary, in this phase Ib trial of intermittent selumetinib in metastatic UM, we found a similar toxicity profile to prior trials involving continuous dosing and determined a MTD of 150mg twice daily. However, this study failed to demonstrate evidence of clinical response with an ORR of 0% and best response of stable disease in 11 patients. We conclude that approaches involving single-agent MEK inhibition in UM lack sufficient utility to support further development, and that further efforts are needed to elucidate mechanisms of clinical response.

## Data availability statement

The raw data supporting the conclusions of this article will be made available by the authors, without undue reservation.

## Ethics statement

The studies involving human participants were reviewed and approved by the Institutional Review Board at each respective institution. The patients/participants provided their written informed consent to participate in this study.

## Author contributions

All authors listed have made a substantial, direct, and intellectual contribution to the work and approved it for publication.

## Funding

Drug and funding for this investigator sponsored research study was provided by AstraZeneca Inc (Wilmington, DE, USA) to support the study. AstraZeneca had no role in data collection, analysis, or interpretation, or in writing this report.

## Conflict of interest

SK reports honoraria from Castle Biosciences. SP reports institutional clinical trial support from Bristol Myers Squibb, Foghorn Therapeutics, Ideaya Biosciences, InxMed, Lvgen, Novartis, Provectus Biopharmaceuticals, Seagen, Syntrix Bio, TriSalus Life Sciences and consulting fees from BMS, Delcath Systens, Advance Knowledge in Healthcare, Cardinal Health, Immunocore, Novartis, and TriSalus Life Sciences. AS reports advisory/personal fees from Bristol-Myers Squibb (BMS), Immunocore, Novartis, and research funding from Pfizer, BMS, Immunocore, Novartis, Targovax, Polaris, Checkmate Pharmaceuticals, Foghorn Therapeutics, Linneaus Therapeutics, and Prelude Therapeutics. GS reports stock or other ownership interests in GenCirq, Bionaut Labs, and January Therapeutics, advisory/consulting/personal fees from Bionaut Labs, Ellipses Pharma, GenCirq, Epizyme, Array BioPharma, Apexigen, Oncogenuity, OnCusp Therapeutics, Concarlo, Shanghai Pharma, Astex Pharmaceuticals, January Therapeutics, Sellas Life Sciences, PureTech Health, and Killys Therapeutics, research funding from Astex Pharmaceuticals, Incyte, Calithera Biosciences, Lilly, Daiichi Sankyo, Fortress Biotech, Karyopharm Therapeutics, Oxford Biotherapeutics, TopAlliance Biosciences, Adaptimmune, Springworks Therapeutics, and TRACON Pharma. RC reports consulting fees from Alkermes, Bristol Myers Squibb, Castle Biosciences, Delcath Systems, Eisai, Jiangsu Hengrui Pharmaceuticals, Ideaya Biosciences, Immunocore, InxMed, Iovance Biotherapeutics, Merck, Novartis, OncoSec Medical, Pierre Fabre, PureTech Health, Regeneron, Sanofi Genzyme, Sorrento Therapeutics, Trisalus and advisory board fees from Aura Biosciences, Chimeron, and Rgenix.

The remaining authors declare that the research was conducted in the absence of any commercial or financial relationships that could be constructed as a potential conflict of interest.

## Publisher’s note

All claims expressed in this article are solely those of the authors and do not necessarily represent those of their affiliated organizations, or those of the publisher, the editors and the reviewers. Any product that may be evaluated in this article, or claim that may be made by its manufacturer, is not guaranteed or endorsed by the publisher.
